# Fluoroscopy-Guided Versus Fluoroscopy-Confirmed Ultrasound-Guided S1 Transforaminal Epidural Injection with Pulsed Radiofrequency: A Prospective, Randomized Trial

**DOI:** 10.5152/eurasianjmed.2023.22265

**Published:** 2023-02-01

**Authors:** Selin Güven Köse, Halil Cihan Köse, Feyza Çelikel, Ömer Taylan Akkaya

**Affiliations:** 1 Department of Pain Medicine, Health Science University Derince Training and Research Hospital, Kocaeli, Turkey; 2 Department of Physical Therapy and Rehabilitation, Sakarya Training and Research Hospital, Sakarya, Turkey; 3 Department of Pain Medicine, Health Science University Dışkapı Yıldırım Beyazıt Training and Research Hospital, Ankara, Turkey

**Keywords:** Interventional ultrasonography, fluoroscopy, epidural injections, pulsed radiofrequency treatment, lower back pain, sacral vertebrae

## Abstract

**Objective:**

The aim of this prospective randomized controlled study was to compare the effectiveness and accuracy of the ultrasound- and fluoroscopy-guided S1 transforaminal epidural injection combined with pulsed radiofrequency in patients with lumbosacral radicular pain caused by S1 nerve involvement.

**Materials and Methods:**

A total of 60 patients were randomized into 2 groups. Patients received S1 transforaminal epidural injection combined with pulsed radiofrequency under either ultrasound or fluoroscopy guidance. Primary outcomes were estimated with Visual Analog Scale scores at 6 months. Secondary outcomes included Oswestry Disability Index, Quantitative Analgesic Questionnaire, and patient satisfaction scores during the 6-month follow-up period and procedure-related variables including procedure time and accuracy of the needle replacement.

**Results:**

Both techniques provided significant pain reduction and functional improvement for 6 months compared to baseline (*P* < .001), without statistical significance between groups at each follow-up point. There was no significant difference in pain medication consumption (*P* = .441) and patient satisfaction scores (*P* = .673) between groups. The fluoroscopy guidance for combined transforaminal epidural injection with pulsed radiofrequency at S1 provided a greater accuracy for the cannula replacement (100%) than the ultrasound (93.3%), without significant difference between groups (*P* = .491).

**Conclusion:**

The ultrasound-guided combined transforaminal epidural injection with pulsed radiofrequency at S1 level is a feasible alternative to fluoroscopy guidance. In this study, we reported that the ultrasound-guided technique resulted in similar treatment benefits including improvement in pain intensity and functionality and reduction in pain medication consumption as those in the fluoroscopy group, while reducing the risk for radiation exposure.

**Main Points**

- Ultrasound-guided combined transforaminal epidural injection with pulsed radiofrequency at S1 level is a feasible alternative to fluoroscopy guidance, while reducing the risk of radiation exposure.
- Ultrasound-guided S1 transforaminal epidural injection combined with pulsed radiofrequency with fluoroscopic confirmation has similar accuracy and efficacy to fluoroscopy alone in patients with chronic low back pain.
- Ultrasound-guided spinal interventions would impact treatment outcomes regarding pain intensity, physical disability, and patient satisfaction for individuals with lower back pain.

## Introduction

Lumbosacral radicular pain is defined as low back pain radiating into the lower extremities in a dermatomal pattern caused by compression or irritation of the nerve root.^1^ Initial treatment options include exercise, physical therapy, psychological programs, and oral medications. Interventional treatment options such as transforaminal, interlaminar, or caudal epidural steroid injections should be considered for cases that do not respond efficiently to conservative approaches for a reasonable period of time.^2,3^

Transforaminal epidural injection (TFEI) as a specific targeted modality offers better treatment outcomes and requires the smallest volume of injectate compared to interlaminar and caudal injections by delivering the therapeutic agent as close as possible to the dorsal root ganglion (DRG).^4-7^ For the management of persistent lumbosacral radicular pain, previous research demonstrated that adjuvant pulsed radiofrequency (PRF) treatment of the DRG improved the treatment outcomes of TFEI compared to TFEI alone.^8-10^ The first sacral (S1) nerve root injection and/or PRF is an effective technique in the management of S1 radicular pain and is usually performed under fluoroscopy (FL).^11,12^

With the increasing use of ultrasound (US) technology in chronic pain medicine, the important role of US guidance in a spectrum of spinal interventions, including lumbar facet injection and sacroiliac injection, is clear and well documented. The US has much superiority over FL, including avoidance of radiation exposure, real-time guidance, direct, and dynamic visualization of surrounding structures.^13,14^ Recently, previous research has described the sonoanatomy of the first sacral foramen and the performance of S1 nerve root block using both the in-plane and out-plane approaches with both optimism and concern.^15-17^ A major concern with US-guided TFEI injections is that US guidance may not always ensure correct needle placement and avoidance of intravascular injection. Previous studies reported that concomitant use of FL or computed tomography (CT) with US is a reliable method to overcome such limitations.^18-20^ Nerve stimulation guidance provides sensation or motor responses corresponding to the dermatome and therefore, identifies the accurate level involved in patients’ radicular pain. Herein, we used US and nerve stimulation guidance to improve the success of needle replacement during US TFEI.

The primary aim of this prospective, randomized controlled study was to compare the effect of US and FL guidance for S1 transforaminal injections in conjunction with PRF treatment on pain intensity in lumbosacral radicular pain caused by S1 nerve involvement. Secondary aims were to compare mean changes in functional disability scores and pain medication consumption and procedure-related outcomes including needle replacement accuracy and procedure time.

## Materials and Methods

### Patients and Randomization

This prospective, randomized controlled trial was conducted after receiving the institutional review board approval (04.10.2021, 121/12). The study was registered at ClinicalTrials.gov (NCT05247892). Patients who gave written informed consent after an explanation of the potential benefits and risks were enrolled in the study between February 2022 and April 2022. The study involved 60 patients, aged between 18 and 80 years, with radicular pain in the lower extremity (scoring ≥ 4 on a Visual Analog Scale [VAS]; for >3 months) secondary to a herniated intervertebral disc resulting in S1 nerve root compression. Following conservative treatments such as oral medications and physical therapy, remission was not adequate. Herniated disk was diagnosed by magnetic resonance imaging, but the posterior longitudinal ligament or annulus fibrosus still covered the herniated nucleus pulposus and had not shed any free fragments. Patients with sacroiliac or facet joint pain depending on the clinical or radiological evaluation, inflammatory and rheumatoid arthritis, psychiatric and neurologic disorders, infection, coagulation disorders, disc pathology at other lumbar levels, previous lumbosacral injections within 3 months, previous lumbar surgery, body mass index (BMI) more than 30 kg/m^2^, anatomical anomalies of the lumbar or sacral spine, allergy to contrast medium, steroids or local anesthetics, and pregnant women were excluded. Subjects in both groups were advised to continue their oral medications during the study period if required.

**Figure 1.**
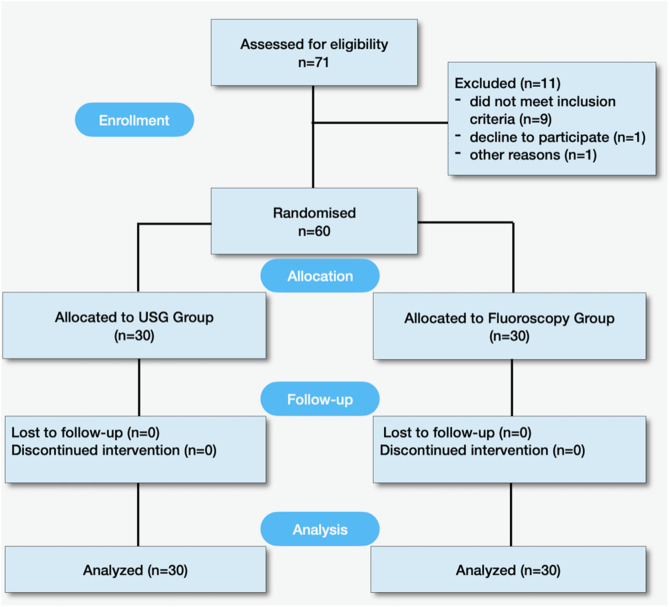
Flow diagram of patients.

The design and process of the study are indicated in the Consolidated Standards of Reporting Trials (CONSORT) diagram (Figure 1). Patients were randomly assigned to receive S1 TFEI in combination with PRF treatment in either the US group or the FL group using a concealed computer-generated randomization protocol with an allocation ratio of 1 : 1. All procedures were conducted by a single pain physician who was not involved in the evaluation process. The interventionist opened the sealed envelope to reveal the treatment assignment for the patient just before the procedure. The randomization process was concealed during the study period from the patients and the investigator who performed all the evaluations. One author, blinded to group allocation and not involved in treatments, performed outcome measurements.

### Interventions

The patient lay in prone position with a pillow under the lower abdomen to align the sacrum horizontally, and an aseptic procedure was done.

### Fluoroscopy Group

Using a fluoroscope, the cephalad-caudad tilt was initially applied to line the L5-S1 endplate. Next, the fluoroscope was rotated at an ipsilateral oblique angle, approximately 5°-15°, until a “Scotty dog” was seen at the L5 vertebral segment. We then found the superomedial landmark of the S1 foramen by drawing an imaginary line down from 6 o’clock position below the L5 pedicle toward the sacral foramen. After skin infiltration with 1% lidocaine, 10 cm, 22 G straight RF cannula with 10 mm active tip was carefully inserted into the respective S1 foramen using intermittent C-arm guidance. Once the RF cannula passed the S1 foramen, a lateral image was obtained to confirm that the needle tip was in the sacral canal and advanced anterior to the ventral border of the sacral canal. The final definite location of the RF cannula required sensory and motor stimulation. The sensory stimulation threshold at 50 Hz should create paresthesia at a voltage of less than 0.5 V and a motor stimulation threshold at 2 Hz of at least 1.5 times the sensory stimulation threshold. Also, the target impedance range of 200-500 Ω was required. The 2-Hz PRF treatment was applied at 45 V, twice for 120 seconds with a small interval in between. During treatment, the electrode tip did not exceed 42°C. The location of the RF cannula was confirmed with the motor/sensory stimulation after the first cycle. After RF procedure, the RF cannula was slightly retreated approximately 1-2 mm to avoid intraneural drug administration. We slowly injected 1 mL of contrast medium and confirmed the location and excluded intravascular uptake using real-time imaging with fluoroscopy. After confirmation of epidural spread using a contrast dye, a 3 mL solution (4 mg of dexamethasone and 0.25% bupivacaine) was slowly injected.

### Ultrasound Group

**Figure 2.**
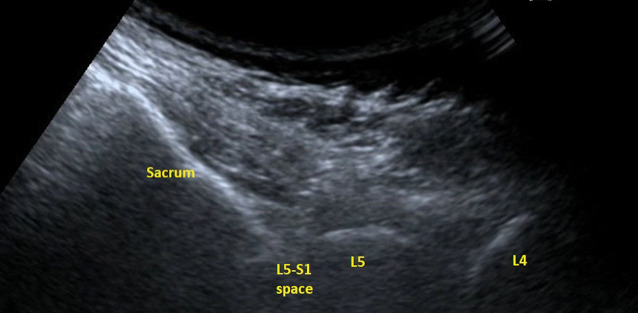
Ultrasound images of the L5/S1.

**Figure 3.**
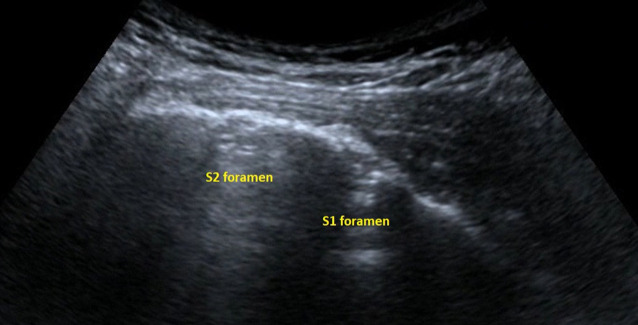
Ultrasound images of the S1 and S2 foramen.

After aseptic preparation, a 2-5 MHz-curved ultrasound probe (GE Healthcare, Wauwatosa, Wis, USA) was placed in the parasacral area, 2-3 cm lateral to the midline, in the longitudinal plane to scan the articular processes of the lower lumbar vertebrae. The articular process was identified at the caudal part of the L5/S1 vertebrae level, and the concavity at the posterior sacral surface located slightly caudally is the S1 posterior sacral foramina (Figure 2). The probe was tilted mediocaudally through the slope of the S1 sacral foramen, showing a hyperechoic area in most cases (Figure 3). An RF cannula was inserted slightly inferolateral to the superomedial oblique using an out-of-plane technique. Although the cannula tip was not visible in the beam, it was possible to estimate its depth from the degree of tissue deformation around the tip. We redirected the needle more mediolaterally when the needle tip came into contact with the posterior sacral surface, and after the cannula passed through the posterior sacral foramen, sensory and motor stimulation was performed to obtain motor and sensory response. Then, the same PRF protocol was performed. To avoid intraneural drug administration, the RF cannula was slightly retreated approximately 1-2 mm. We slowly injected 1 mL of contrast medium and confirmed the location and excluded intravascular uptake using real-time imaging with FL. In the case that the contrast spread was not intraforaminal in the first attempt, the RF cannula was repositioned. After confirmation of dye spread to the epidural space in the anteroposterior and lateral images and with sensory/motor stimulation, a 3 mL solution (4 mg of dexamethasone and 0.25% of bupivacaine) was slowly injected.

### Outcome Measurements

Descriptive data, including age, gender, body mass index, side of the procedure, and duration of pain, were collected at baseline. The 100 mm VAS score was calculated on a scale from 0 (no pain) to 100 (worst imaginable pain).^21^ While the primary outcome was pain intensity, secondary outcomes were the rate of successful responders (reduction in VAS score by at least 50% at 6 months after the procedure compared with baseline), mean changes in functional disability scores and pain medication consumption, changes in patient satisfaction, and procedural-related variables.

The Oswestry Disability Index (ODI) is a self-administered questionnaire consisting of 10 items of functional ability, each with 6 options ranging from 0 to 5. The percentage of disability, the total ODI score, is obtained using the equation: total score/50 × 100. While 0% represents no pain or disability, 100% represents the most severe pain and disability.^22^ Visual Analog Scale and Oswestry Disability Index were evaluated at baseline, and at 2 weeks, 1, 3, and 6 months after the procedure.

Participants’ overall satisfaction was assessed at 6 months using a 5-point Likert scale (1, very dissatisfied; 2, dissatisfied; 3, neutral; 4, satisfied; 5, very satisfied).^23^ Mean change in analgesic consumption was assessed at 6 months using Quantitative analgesic questionnaire (QAQ), a tool designed to record patient-reported pain medication use, create scores to quantify and compare it, and track changes in analgesic drug use over time. A higher score indicates higher pain medication use.^24^

Procedural-related variables included procedure time and accuracy of the cannula replacement at the first attempt. Procedure time was measured using a stopwatch. It was defined as the time from the beginning of the procedure, the initial image was obtained, until the end of the procedure when: the S1 root was clearly seen when 1 mL of contrast medium was injected and the investigator stated satisfaction with the image findings.

### Statistical Analysis

Statistical analyses were performed using the statistical analysis program International Business Machines’ Statistical Package for Social Sciences version 16.0 (SPSS Inc.; Chicago, IL, USA). The Shapiro–Wilk test was used to test the assumption of normality. Continuous variables, normally distributed, were presented as mean and standard deviation and continuous variables without normal distribution, median (interquartile range). The independent sample *t* test or the Mann–Whitney *U* test was used for the comparison of continuous variables. Categorical data were presented as counts and percentages and compared by chi-square test or Fisher’s exact test (when 25% or more cells had expected counts of less than 5). A 2-way repeated measures analysis of variance with group as a factor within subjects, and time (between baseline and follow-up assessments) as a factor within subjects was used to detect significant differences in the outcome measure scores within and between the 2 groups, with post hoc Bonferroni tests for multiple comparisons. A value of *P* < .05 was considered statistically significant.

Sample size calculation was performed using G*Power software version 3.1.9.7 (Heinrich-Heine-Universität, Düsseldorf, Germany) according to our preliminary study data. In our clinic, we conducted a preliminary study of 10 patients with a mean (± standard deviation [SD]) VAS score of 3.5 ± 1.4 for FL-guided S1 TFEI in combination with PRF treatment at 6 months. Using our preliminary results and considering the VAS as the primary outcome at month 6, a sample size of 25 patients in each group was determined to be necessary in order to detect a 30% between-group difference and a level of .05, and power of 80%. Considering a 15% dropout probability, we included 30 patients in each group.

## Results

### Study Population

Sixty patients who met the inclusion criteria were included in the study and randomized with allocation into 2 treatment groups. The patients’ baseline demographic data and clinical characteristics were similar between the treatment groups. There was no significant difference between the groups in terms of gender, age, body mass index, disease duration, previous surgery, and injection side (*P* > .05) (Table 1). No block-related complications were reported in any patient during the follow-up period.

There were no significant differences between the groups in the VAS (F (1.58) = 1.584, *P = .*213) and ODI (F (1.58) = 0.622, *P = .*434) scores at baseline and each follow-up point after the procedures. There was no significant interaction between time and group allocation for the VAS (F (4.55) = 0.564, *P = .*690) and ODI (F (1.55) = 1.17, *P = .*330) scores. In both groups, a significant effect of time was found in VAS (F (4.55) = 352.165, *P < .*001) and ODI (F (4.55) = 72.9, *P < .*001) scores. Visual Analog Scale values decreased significantly in both treatment groups, with sustained treatment effects at all time points compared to baseline (*P < .*001) (Table 2). Significant improvements were observed in ODI scores in both groups during the 6 months follow-up period compared to baseline scores (*P < .*001) (Table 2). The ratio of patients who experienced at least 50% pain reduction was similar at each follow-up point. At 6 months after the procedure, 43.3% (13/30) of patients in the FL-guided group compared with 46.6% (14/30) in the US-guided group had positive results with respect to these successful treatment outcome criteria (Table 3).

At 6 months, a significant reduction in pain medication consumption was observed in both groups compared to baseline (*P < .*001). On the 5-point Likert scale, 50% and 56.7% of the patients were very satisfied or satisfied with treatment in the US and FL group, respectively. However, no significant difference was present in QAQ and patient satisfaction scores between the US and FL groups (*P* > .05) (Table 2).

In the US-guided group, 28/30 (93.3%) procedures were completed successfully at the first attempt, while all the procedures were successful at the first attempt in the FL group. There was no significant difference regarding the procedure-related outcomes including procedure time and accuracy of the needle replacement at the first attempt (*P* > .05) (Table 4).

## Discussion

This prospective, randomized controlled study was designed to compare the US and FL guidance for treatment and procedure-related outcomes of S1 TFEI in conjunction with PRF treatment in patients with S1 radicular pain secondary to disc herniation. The results of the present study suggest that the US-guided technique is a convenient and safe alternative treatment modality, with reduced radiation exposure. When comparing these 2 therapeutic groups, there was no difference in treatment outcomes, including pain and functional disability scores, patient satisfaction, and pain medication use, as well as procedure-related outcomes including needle replacement accuracy and procedure time.

Recently, US imaging has emerged as an alternative method to guide spinal interventional techniques. Ultrasound guidance provides compelling evidence supporting its use in sacroiliac joint, caudal epidural, and facet joint injections, and medial branch nerve blocks.^13,14,25^ Many studies comparing US- and FL-guided techniques for those procedures found similar improvement in pain relief and functional disability and overall patient satisfaction scores, as well as no difference in number of needle passes, complications, and adverse events.^26-28^ However, lumbosacral transforaminal injections under US guidance are still debatable among interventional pain specialists due to concerns that US may not ensure correct needle placement.^29,30^ Therefore, a prospective randomized study comparing the accuracy of needle replacement, effectiveness, and safety of US guidance with FL was necessary to address lacunae in literature.

To our knowledge, this is the first study to utilize both treatment success and procedure-related outcomes to assess US and FL guidance for S1 TFEI in combination with PRF treatment in patients with radicular pain. Both approaches yielded similar success rates in terms of treatment outcomes and resulted in similar accuracy rates for needle replacement and procedural times. Based on the findings of this study, we suggest that it is reasonable to consider the use of US as an alternative to FL. Supportingly, there is growing evidence that the US is a reasonable alternative to FL for TFEI. In one study, Loizides et al^19^ compared lumbar US-guided TFEI with the CT-guided technique, and another study compared lumbar US-guided TFEI with the FL technique.^20^ Both studies demonstrated that US guidance shortened procedure time and resulted in a reduction in radiation dose with the same benefits. In terms of needle replacement accuracy, the results found in this trial were slightly higher than the results of previous studies that reported success rates of approximately 85% and 90% for needle replacement in US-guided lumbar TFEI.^19,20^ Unlike other levels of the lumbar spine, the first sacral foramina are easily found under US guidance due to its proximity to the skin. Supportively, a cadaveric anatomical study evaluating the accuracy of the S1 TFEI revealed that US guidance provided 100% accuracy of needle placement.^15^

In addition to US or FL guidance, the application of PRF adjacent to the DRG requires sensory stimulation inducing paresthesia consistent with the existing distribution of the nerve root dermatome to confirm the RF cannula position. Although the generation of radiating pain or paresthesia is not always necessary in the TFEI procedure, we propose that the use of electrical nerve stimulation guidance may improve the success of needle trajectory for US-guided procedure and correct target positioning, as well as reduce the risk of direct nerve injury.

Regarding pain relief, the efficacy of TFEI combined with PRF observed in this study is similar to the result of previous studies that reported a success rate between approximately 60% and 80% in patients with lumbar radicular pain at 3 months.^10,31,32^ Of the prior studies that investigated the effect of adjuvant PRF to TFEI, there was a significant improvement in treatment outcomes in TFEI and PRF combination when compared to TFEI alone. In a recent study, Ding et al^8^ demonstrated that PRF combined with TFEI achieved a better clinical outcome and long-term remission of lumbar disc hernia than PRF alone. In another study, Koh et al^9^ reported that TFEI applied in conjunction with PRF achieved better treatment success than TFEI alone in patients with lumbosacral radicular pain due to spinal stenosis after 2 and 3 months, but there was no difference in outcome after 1-month follow-up. In a recent trial, Caliskan et al^10^ demonstrated that the application of DRG-PRF in addition to TFEI provided a significant improvement in pain intensity compared to TFEI alone in patients with chronic lumbar radicular pain at any time point during the 3-month follow-up period.^10^ In our clinical practice, adjuvant PRF therapy was routinely used to prolong the duration of TFEI and improve treatment outcomes. Our results suggest that both US- and FL-guided S1 TFEI in combination with PRF treatments are safe, efficient, and applicable in patients with S1 radicular pain who do not respond to conventional therapy.

With regard to procedure time, the results of this study showed no difference between these 2 approaches. The use of FL is recommended to confirm the accurate needle location until physicians have enough experience with the US approach.^15,18,28^ Thus, the additional time needed for US-guided TFEI injection with fluoroscopic confirmation and some radiation exposure must be taken into account, particularly for more novice users. Relief of symptoms including improvement in pain relief or functional disability is the gold standard for assessing the success of interventional techniques. Even if the RF cannula was not accurately placed in the target area, symptoms may be relieved by steroid injection spreading to the DRG, leading to inaccurate estimation rates. We investigated accuracy by confirming the placement of the needle tip and the spread of the radiocontrast agent with FL.

Our study has a few limitations. First, the trial was not conducted as a double-blinded, controlled study. With techniques such as US or FL, conducting a double-blinded, controlled trial is challenging. Second, 1 interventionist with experience in those techniques performed all procedures in this study, which may limit the generalizability of the results. Third, only patients with a BMI < 30 kg/m^2^ were included in this trial. It is technically challenging to apply US guiding for spinal interventions in individuals with higher BMI, and future research is required to determine whether US can be used for S1 TFEI in this population. Finally, FL guidance was used to ensure proper needle placement and detection of intravascular placement. Thus, some radiation exposure must be taken into account.

In conclusion, the US-guided combined TFEI and PRF at S1 level is a feasible alternative to FL guidance in patients with lumbosacral radicular pain secondary to S1 nerve involvement. In this study, we reported that the US-guided technique provided similar treatment benefits including improvement in pain intensity and functionality and reduction in pain medication consumption compared with the FL group. We suggest that the S1 TFEI combined with PRF meticulously performed under hybrid US/FL guidance may be used as a valuable technique in therapeutic procedures, with reduced radiation hazard.

## Figures and Tables

**Table 1.** Baseline Characteristics of Patients and Clinical Variables

Characteristics	US Group (n = 30)	FL Group (n = 30)	*P*
Age, years	54.26 ± 10.26	55.96 ± 11.22	.542
Sex, n (%)			
Female	10 (33.3)	11 (36.6)	.794
Male	20 (66.6)	19 (63.4)	
BMI, kg/m^2^	25.82 ± 2.69	25.3 ± 2.41	.433
Duration of pain, months	23.8 ± 9.82	25.83 ± 13.5	.508
Injection side, n (%)			
Left	13 (43.3)	15 (50)	.796
Right	17 (56.7)	15 (50)	
Previous surgery	6 (20)	5 (16.6)	.753
VAS score	6.96 ± 0.85	6.56 ± 1.19	.139
ODI score	49.46 ± 13.85	43.93 ± 13.92	.128
QAQ	3.30 ± 1.17	3.46 ± 08	.534

Values are expressed as the mean ± standard deviation or number of patients (%).

BMI, body mass index; FL, fluoroscopy; ODI, Oswestry disability index; QAQ, Quantitative analgesic questionnaire; US, ultrasound; VAS, visual analog scale.

**Table 2.** Follow-Up of Study Scales in Both Groups

	US Group		FL Group		*P*
	Mean Score (95% CI)	Cohen’s *d*	Mean Score (95% CI)	Cohen’s *d*	Group Comparison
VAS					
Baseline	6.96 ± 0.85 (6.64-7.28)		6.56 ± 1.19 (6.12-7.01)		.140
2 weeks	2.93 ± 0.9* (2.59-3.27)	4.56	2.6 ± 0.89* (2.26-2.93)	3.76	.157
1 month	3.13 ± 1.07* (2.73-3.53)	3.96	2.93 ± 0.94* (2.54-3.25)	3.38	.379
3 months	3.6 ± 0.81* (3.29-3.9)	4.04	3.33 ± 1.06* (2.93-3.72)	2.86	.279
6 months	3.9 ± 0.84* (3.58-4.21)	3.62	3.8 ± 1.12* (3.37-4.22)	2.38	.699
ODI					
Baseline	49.46 ± 13.85 (44.29-54.64)		43.93 ± 13.92 (38.73-49.13)		.128
2 weeks	21.93 ± 8.05* (18.92-24.94)	2.43	22 ± 6.74* (19.48-24.51)	2.41	.972
1 month	17.8 ± 6.39* (15.41-20.18)	2.93	17.6 ± 6.31* (15.24-19.95)	2	.903
3 months	24 ± 5.4* (21.98-26.01)	2.42	23.13 ± 6.92* (20.54-25.71)	1.89	.591
6 months	30.6 ± 8.22* (27.52-33.62)	1.65	29.93 ± 9.41* (26.41-33.44)	1.17	.771
QAQ					
Baseline	3.30 ± 1.17 (2.85-3.74)		3.46 ± 08 (3.14-3.78)		.534
6 months	1.50 ± 09 (1.16-1.83)	1.72	1.76 ± 0.67 (1.51-2.02)	2.30	.201
Patient satisfaction					
6 months	3.5 (3-4)		4 (3-4)		0.673

Values are expressed as the mean ± standard deviation, median (interquartile range).

^*^ *P < .*001 is considered statistically significant according to baseline.

FL, fluoroscopy; ODI, Oswestry disability index; QAQ, Quantitative analgesic questionnaire; US, ultrasound; VAS, visual analog scale.

**Table 3.** At least 50% Decrease in VAS Score

	US Group		FL Group		*P*
	n	%	n	%	
2 weeks	24	80	25	83.3	.739
1 month	22	73.3	25	83.3	.347
3 months	21	70	23	76.6	.559
6 months	14	46.6	13	43.3	.795

Values are presented as numbers (percentage).

FL, fluoroscopy; US, ultrasound; VAS, Visual Analogue Scale.

**Table 4.** Procedure-Related Outcomes

	US Group	FL Group	*P*
Procedure time (seconds)	353.16 ± 42.31	364.53 ± 53.98	.367
Accuracy	28 (93.3%)	30 (100%)	.491

Accuracy, accuracy of the needle replacement at the first attempt; FL, fluoroscopy; US, ultrasound.
